# From Concept to Practice: Lessons From the Balanced Nursing Teams Decision-Support System

**DOI:** 10.2196/92417

**Published:** 2026-05-11

**Authors:** Senne Vleminckx, Peter Van Bogaert, Wim De Keyser, Filip Haegdorens

**Affiliations:** 1 Workforce management, Health systems, and Outcome Research in Care – Centre for Research and Innovation in Care, Faculty of Medicine and Health Sciences, University of Antwerp, Universiteitsplein 1, Wilrijk, Antwerp, 2610, Belgium, 32 474859762; 2 Applied Computer Science, Sustainable Industry Research Team, Karel de Grote Hogeschool, Antwerp, Flanders, Belgium

**Keywords:** nursing workforce management, clinical decision-support systems, implementation science, sociotechnical barriers, nursing leadership

## Abstract

The global nursing workforce crisis demands a shift from reactive staffing to strategic workforce optimization through data-driven decision-support systems. This viewpoint paper reflects on the development and attempted implementation of the balanced nursing teams system, a decision-support tool integrating approximately 250 data points—of which roughly 150 are extracted from existing organizational systems (human resources, scheduling, electronic health records, quality registries) through flexible import mechanisms, and the remainder collected through a built-in 360-degree staff survey with automated analysis—across 10 domains to evaluate nursing team balance between capacity, performance, and outcomes. Following crowdfunding by 18 Belgian health care organizations, balanced nursing teams were implemented across 8 diverse settings (home health care, general hospitals, academic centers) between 2019 and 2023. Using the Human-Organization-Technology fit framework, we analyze why evidence-informed, organization-endorsed digital innovations struggle to achieve adoption. Our analysis reveals 3 interdependent barrier categories: technological fragmentation (vendor lock-in, legacy systems, prohibitive integration costs), organizational siloing (Chief Nursing Officers [CNOs] lacking budgetary authority, nursing framed as peripheral to strategic priorities), and managerial hesitance (fear of punitive data use, cognitive overload from staffing crises). These barriers were worsened by the substantial data-integration burden that the system’s breadth imposed on organizations with limited digital maturity. Critically, only one site (ie, a nurse-led home health care organization where leadership held both strategic authority and resource control) achieved sustained implementation. This contrast demonstrates that workforce optimization through data depends not on software maturity alone, but on achieving simultaneous fit across human, organizational, and technological domains. We argue that the persistent marginalization of nursing leadership within hospital governance structures represents the fundamental barrier to digital transformation in nursing workforce management. The urgency paradox is striking: while nursing represents health care organizations’ highest operational cost and most direct patient interface, workforce optimization tools are consistently deprioritized in favor of regulatory compliance systems and billing infrastructure. Bridging this gap requires systemic investment in nursing leadership authority, data interoperability standards, and recognition that data-driven workforce decisions are strategic imperatives rather than operational luxuries.

## Introduction

### Overview

The global nursing workforce crisis extends beyond simple shortages. Persistent mismatches between staffing levels, skill mix, and patient demand have been linked to missed care, adverse outcomes, and failure to rescue, ultimately compromising both patient and staff outcomes [1-4]. While policymakers have focused primarily on increasing the number of nurses entering the workforce, evidence indicates that expanding supply alone does not resolve the underlying staffing challenges [5,6]. The retention of experienced nurses depends on the work environment quality and team performance [7], yet high turnover continues to impose substantial financial and operational burdens on health care organizations [8].

Current staffing models, such as mandatory nurse-to-patient ratios, fail to capture the complexity of nursing team dynamics, skill mix optimization, and broader contextual factors that determine whether care teams can effectively meet patient demands [9]. Addressing this challenge requires a shift from ratio-based workforce planning towards data-informed team design that integrates human, organizational, and contextual dimensions of nursing care [9].

This viewpoint paper offers a critical reflection on the sociotechnical challenges of implementing digital workforce innovation in nursing. Drawing from our experience developing and testing the balanced nursing teams (BNuT) decision-support system across 8 Belgian health care organizations, we examine why evidence-informed, practitioner-endorsed digital tools struggle to achieve real-world adoption. Rather than presenting a traditional implementation study, we provide an analytical perspective on the systemic barriers that constrain digital transformation in nursing workforce management, offering lessons for researchers, health care organizations, and policymakers pursuing similar innovations.

### A Nursing Teams Composition and Staffing Decision-Support System: BNuT

In response to these challenges, the BNuT initiative was launched in 2018 through a collaboration between a member organization of Belgian health care organizations and 2 research groups. The project originated from a clear practical demand: develop an instrument that enables evidence-based, proactive, and data-driven decisions regarding nursing team composition. Following proof-of-concept development, 18 health care institutions collectively crowdfunded the project, reflecting broad recognition of this critical gap in nursing workforce management.

**Figure 1.**
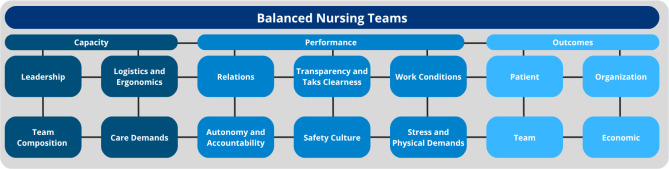
Conceptual model of the balanced nursing teams (BNuT) system. The figure depicts the 10 subcomponents supporting the BNuT decision-support system, organized into 2 overarching categories: capacity domains (team composition, care demands, staffing, and skill mix) and performance domains (leadership, logistics, safety culture, workload distribution, team dynamics, and professional development). These domains collectively inform the assessment of team balance, ranging from negative imbalance through optimal balance to positive imbalance.

BNuT was conceived as a computerized decision-support instrument designed to optimize team composition and team functioning in ways that existing tools cannot. While most workforce software estimates patient demand or calculates staffing levels based on mandatory ratios [10], these systems rely heavily on manual governance and contextual adjustment. In contrast, BNuT integrates data across multiple domains—including relational dynamics, autonomy, care demand, leadership, logistics, and safety—into a unified team-level model of workforce balance (Figure 1).

The system estimates team balance on a continuum ranging from negative imbalance (eg, excessive workload and high risk of burnout) through optimal balance to positive imbalance (eg, over-capacitated teams and high risk of boreout). This continuum builds on earlier conceptualization of a balanced care team as one in which capacity, including care demands and team composition (4 domains) and operational performance processes (6 domains), is strategically aligned to optimize outcomes for both staff and patients, as well as organizational and economic outcomes [9]. Sustained imbalance between the 10 domains undermines both care quality and workforce sustainability. BNuT aims to operationalize this principle by assessing the alignment between nursing workforce organization and patient care needs, translating complex multidimensional data into actionable, evidence-based visual indicators for team and managerial decision-making.

The development of BNuT followed a staged approach designed to progressively establish validity and relevance. The underlying model was empirically grounded in a systematic scoping review that identified 35 factors across 9 domains of balanced team functioning [9]. The indicator set was subsequently refined through structured codevelopment with nursing managers from participating institutions, who assessed each variable’s clinical importance, data availability, and interpretive complexity. The application itself was co-designed with end users through user-story workshops that shaped functionality, interface design, and workflow integration. This iterative process established content validity, ensured practical relevance, and confirmed usability. However, criterion validation against patient and staff outcomes, which was originally planned via the operational deployment in participating hospitals, could not be completed. This validation gap was caused by the sociotechnical barriers described in this paper, as they prevented the successful implementation necessary for outcome evaluation. BNuT should therefore be understood as a theoretically and empirically informed decision-support prototype. Its outcome validation remains contingent on achieving the organizational conditions for sustained use.

### Aim and Analytical Framework

This paper provides a reflective analysis of the sociotechnical challenges encountered during the development and testing of BNuT. We move beyond technical specifications to examine why digital innovations supported by both evidence and organizational demand struggle to achieve real-world adoption. Our analysis uses the Human-Organization-Technology fit (HOT-fit) framework [11], a model specifically developed for evaluating health information systems. This framework maps the 3 principal barrier categories observed in our work and highlights how interactions among human, organizational, and technological factors jointly shape implementation outcomes. Each dimension is examined systematically, followed by an integrative reflection demonstrating how compounding barriers can obstruct implementation even when individual system components function as designed. This viewpoint does not present outcome validation of the BNuT system; rather, it offers a structured reflective analysis of implementation dynamics, informed by 8 years of engagement with participating organizations and stakeholders across the Belgian health care field.

The insights presented in this viewpoint are grounded in 8 years of sustained engagement with the BNuT project (2018‐2026), encompassing the full cycle from conceptualization and codevelopment to attempted implementation across 18 health care institutions. During this period, the author team engaged in extensive, iterative dialogues with stakeholders across all levels of the health care system (eg, frontline nurses, nursing managers, CNOs, data managers, data protection officers, IT departments, and external development partners) through codevelopment workshops, pilot testing sessions, steering committee meetings, and formal presentations to prospective adopters and investors. Each of these interactions generated feedback that progressively shaped the team’s understanding of the barriers and enablers discussed in this paper. The reflections synthesized here emerged iteratively from this long-term, multi-stakeholder engagement. This approach prioritizes ecological breadth and depth of immersion over the rigor of structured qualitative research. The HOT-fit framework [11] was applied retrospectively to organize and analyze these accumulated insights, providing an analytical foundation.

## The BNuT System and Implementation Context

### BNuT Concept and Architecture

Health care organizations face a fundamental data paradox in nursing workforce management. While data seems abundant, the information remains siloed across systems, leaving leaders without the integrated insight required for strategic workforce decisions. Existing information systems were primarily designed for billing and regulatory compliance rather than operational or strategic decision-making. As a result, critical data concerning team dynamics, workload distribution, competency mix, and patient demand remain fragmented across incompatible platforms, such as electronic health records (EHRs), scheduling tools, human resources (HR) databases, and quality registries. This fragmentation and the lack of comprehensive data warehouses or business intelligence tools leave nurse managers operating largely reactively, addressing daily staffing shortages without insight into long-term workforce patterns or their impact on safety, quality, and retention. Moreover, staff lack a data-informed understanding of their team capacity, performance, and outcomes, and must rely largely on perceptions. This data paradox has become increasingly consequential as contemporary health care systems face mounting pressures: cost containment imperatives, heightened quality and safety demands, and growing clinical hyperspecialization that requires more sophisticated approaches to team composition and skill-mix optimization.

BNuT aims to address these challenges through 2 interconnected objectives that shift workforce management from reactive to strategic. The first involves comprehensive data collection and processing to enable both cross-sectional analyses (capturing current team states) and longitudinal tracking (monitoring team evolution). This supports standardized decision-making capable of distinguishing between expected variance, acceptable fluctuations, and disruptive imbalances. The second objective focuses on integrating multiple data streams into meaningful composite indicators at the clinical microsystem level, encompassing workload intensity, competency distribution, leadership effectiveness, safety climate, and related domains to estimate the balance between team capacity, performance and care demands, and outcomes.

To achieve this, the system combines a 360-degree evidence-based survey, capturing staff perceptions on statements within each capacity and performance domain, and data drawn from existing organizational systems such as scheduling software, EHRs, and quality metrics. All data are synthesized using established evidence-based standards in nursing workforce research, ensuring that the resulting indicators reflect empirically supported relationships between the domains. Crucially, the unit of analysis is the clinical microsystem, acknowledging that patient and team outcomes emerge from collective team processes rather than individual performance metrics. The system supports dynamic management through continuous monitoring and benchmarking functions, allowing leaders to compare performance across teams, identify high-performing configurations, and support continuous improvement. Team involvement in discussing these data is paramount to support teams and leaders in understanding team strengths and weaknesses, to identify improvement priorities, and to assess the extent of readiness for innovation [12]. BNuT thus provides managers and leadership with both a helicopter view for strategic oversight and the ability to zoom in on specific teams or metrics when deeper investigation is required, thereby supporting decision-making at operational, tactical, and strategic levels.

### Implementation Setting and Challenges

BNuT was implemented across 8 health care organizations representing diverse contexts, including home health care, general hospitals, and academic centers. Although the degree of adoption varied, each site revealed distinct yet interrelated sociotechnical barriers that influenced feasibility and uptake:

1. At the human level, hesitance and abstention emerged as key barriers. While these responses may originate as reactions to perceived risks, the resulting behavioral patterns—reluctance to engage, delayed adoption, or outright avoidance—function as implementation barriers. Nursing leaders and managers expressed concern that BNuT-generated insights might be misused in their performance evaluations. There was also apprehension about receiving feedback without having the resources or organizational support to act on it, creating a sense of accountability without agency. The operational pressures of managing day-to-day staffing crises left little cognitive bandwidth for strategic workforce optimization. This environment discouraged engagement with reflective data tools, despite general support for the underlying vision.
2. At the organizational level, siloed priorities and hierarchical governance structures undermined implementation. Financial executives, facing severe budget constraints (particularly relevant given that approximately 40% of Belgian hospitals operate at a financial loss [13,14], perceived workforce optimization tools as an additional cost rather than an investment. Chief technology officers, while conceptually supportive, found it difficult to justify allocating scarce IT resources to early-stage, nurse-led projects lacking immediate financial return. This division between nursing leadership (operational authority) and executive governance (strategic control) created a structural misalignment that limited implementation feasibility.
3. At the technological level, integration of existing systems proved prohibitively complex and expensive due to vendor lock-in strategies, legacy system fragmentation, and deliberate data walling between platforms. Several hospitals had outsourced IT operations, transforming what could have been internal configuration tasks into costly external service requests requiring formal budget approval. IT departments, already constrained by workforce shortages and competing project portfolios, often struggled to prioritize nurse-led digital innovations.

Out of the 8 health care organizations, one site achieved substantial success offering a contrasting perspective. Operating as a nurse-led home health organization, its leadership culture embedded the vision of data-driven workforce optimization from the outset. Alignment between operational need, executive vision, and organizational authority enabled integration of BNuT into management routines. This contrast underscores the critical role of organizational alignment and leadership ownership in realizing the potential of sociotechnical innovations such as BNuT.

## The 3 Dimensions of Barriers

### Identifying the HOT-Fit

**Figure 2.**
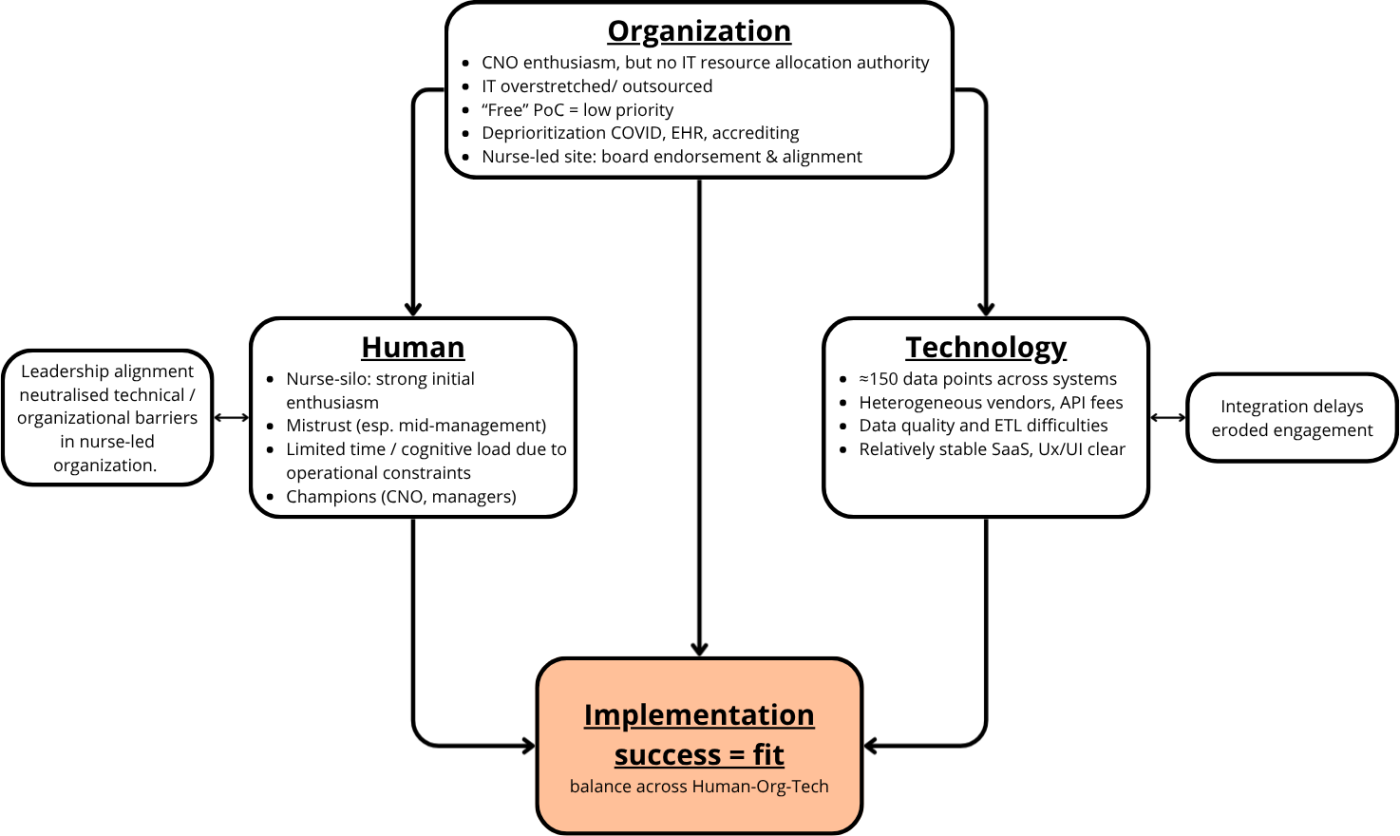
HOT-fit framework applied to the BNuT implementation. The diagram illustrates the 3 interacting domains of the HOT-fit model and their relationships in the context of the BNuT decision-support system. Arrows represent interdependencies: successful implementation (fit) occurs only when all domains are simultaneously aligned. Callouts highlight two key observations: (1) strong fit within the organizational domain—where nursing leadership held both strategic authority and operational responsibility—enabled the nurse-led organization to overcome technical barriers, and (2) data-integration delays eroded user engagement. API: application programming interface; BNuT: balanced nursing teams; CNO: chief nursing officer; EHR: electronic health record; ETL: extract-transform-load; HOT: Human-Organization-Technology; PoC: proof of concept; SaaS: software-as-a-service; UX/UI: user experience/user interface.

The implementation experiences of the BNuT project were examined through the HOT-fit framework [11]. The framework provides a sociotechnical lens for analyzing how system adoption and sustainability depend on the alignment, or fit, between 3 interacting domains: human, organizational, and technological. Our reflections demonstrate that BNuT’s implementation challenges were not isolated to any single dimension. Rather, success depended on achieving an equilibrium across domains, and an imbalance in one frequently undermined progress in the others (Figure 2). This section reflects on lessons learned within each domain and concludes by considering the interdomain dynamics that shaped implementation outcomes.

### Human: Trust, Empowerment, and Cognitive Capacity

Across the participating organizations, nursing leaders were consistently enthusiastic about BNuT’s conceptual promise. The idea of a data-driven instrument capable of translating complex workforce information into actionable team-level insights resonated strongly with their daily struggles. Notably, the project itself originated from nursing leaders’ explicit requests for better decision support tools, making BNuT a response to practitioner demand rather than a top-down research initiative. Early skepticism during prototype demonstrations largely dissipated once realistic use cases were presented. The urgency of developing such a system was widely acknowledged, and many leaders expressed relief that a project finally addressed the persistent gap between workforce research and managerial decision support.

*The added value that this tool will be able to give us in the future was clear to everyone in the meeting. It will enable us to make an in-depth analysis based on objective data about the functioning of a team and also indicate on which elements action is required to remedy this*.

[Chief Nursing Officer, participating hospital]

*BNuT provides a most powerful lens for every nurse manager. Gain personal leadership and team insights to transform your department with data-driven decisions for better care and satisfied staff*.

[Nurse Manager involved in BNuT pilot testing]

However, this enthusiasm coexisted with overconfidence regarding implementation feasibility. Many nursing leaders underestimated the organizational and technical barriers involved and overestimated their own authority to overcome them. Hospitals with advanced data infrastructures were paradoxically more hesitant, perceiving BNuT as “yet another system” rather than as a complement to existing business-intelligence tools. These organizations typically requested an Application Programming Interface (API) to integrate BNuT’s algorithms into their own dashboards rather than adopting the full platform. Conversely, smaller organizations, particularly those without internal analytics capacity, perceived BNuT as uniquely valuable but lacked the resources to integrate it.

Hesitance surrounding data use proved a recurrent issue, though its nature differed markedly across organizational levels. Among frontline nurses, concerns were minimal once the survey’s anonymity was assured; they generally perceived BNuT as a supportive and developmental tool rather than a threat. In contrast, hesitance and some mistrust were concentrated among junior and mid-level managers, who feared that aggregated results, particularly those reflecting leadership climate or organizational support, could be interpreted punitively by higher management. In one large university hospital, even the CNO voiced concern that unfavorable scores on *“*organizational leadership*”* might be politically exploited by other executives. These perceptions reveal the asymmetrical distribution of vulnerability within hierarchical hospital structures: while staff trusted the system’s safeguards, middle management occupied a position where performance data could plausibly affect professional standing.

Demonstrations and early pilots substantially increased perceived usefulness. Once users saw how disparate data streams converged into interpretable insights, engagement improved markedly. A frequently cited example involved incident reporting: one team appeared to have a disproportionately high number of reported safety incidents. Initially interpreted as poor performance, the BNuT analysis revealed that this team simultaneously scored highest on safety culture, indicating a healthy incidence-reporting climate. Such cases illustrated how integrated data interpretation could challenge misperceptions and reframe organizational learning.

Despite these successes, cognitive and temporal constraints limited sustained engagement. Nursing managers facing chronic staffing shortages and operational crises had little time to interact with a strategic-level dashboard. The tool’s intuitive design mitigated digital-literacy barriers, yet the absence of formal onboarding and the overall cognitive load of crisis management restricted deeper adoption. Champions, typically CNOs or middle managers, emerged in nearly all sites, but their influence was constrained by limited institutional authority. Over time, enthusiasm at the human level could not compensate for systemic resource constraints and organizational inertia.

However, it must also be acknowledged that the system’s own scope likely contributed to the cognitive and governance burden experienced by potential adopters. BNuT encompasses 10 domains and integrates approximately 250 data points, of which roughly 150 are to be extracted from organizational systems. While the user-facing interface was specifically designed to simplify interpretation by reducing this complexity to composite indicators and visual summaries, the underlying data requirements imposed demands on organizational processes that were substantial, particularly for institutions already operating under severe resource constraints. For organizations with limited digital maturity, the gap between the system’s data needs and the institution’s capacity to deliver that data may have been too large, regardless of the tool’s analytical value. In retrospect, a more modular deployment strategy might have lowered the threshold for initial adoption: beginning with a reduced core indicator set drawn from readily available data and progressively activating additional domains as organizational data capacity matured would have allowed trust, familiarity, and demonstrable value to develop incrementally before the full model was introduced.

### Organizational: Leadership Alignment and Structural Inertia

The organizational context proved decisive. In all hospitals, the CNOs were strong advocates for BNuT, yet they lacked budgetary control. IT departments, already overstretched and managing numerous competing projects, had to allocate technical resources, while financial decisions ultimately rested with chief financial officers. Because the project was offered free of charge during the proof-of-concept phase, it competed poorly for attention against commercial initiatives with contractual obligations. In hindsight, requesting a modest financial buy-in might have enhanced commitment and prioritization by signaling ownership.

Both HR and IT departments often viewed BNuT as redundant. HR staff perceived it as encroaching on their existing tools, while IT departments regarded it as a low-priority research experiment. Chronic shortages of IT personnel further reduced their willingness to support time-consuming integrations. In several hospitals, IT services had been partially outsourced, transforming even minor configuration tasks into expensive external contracts. In some cases, hospitals lacked in-house capacity to access their own databases, as software vendors maintained exclusive control and charged high fees for basic queries. These arrangements effectively held data “hostage” and created a very high dependency on vendors for data extraction.

Organizational fit, the degree to which nursing leadership held authority corresponding with operational responsibility, emerged as the critical differentiator. In hierarchical organizations where the nursing department functioned as a silo, CNOs struggled to gain board-level approval. Projects framed as nurse-led were perceived as peripheral to strategic objectives. By contrast, the one nurse-led organization integrated BNuT into its formal strategic plan, supported by executive leadership and dedicated IT allocation. This fit between operational need, strategic vision, and resource authority was essential. It enabled the project to progress from conceptualization to sustained use, highlighting the necessity of structural empowerment for nursing leadership within governance systems.

*We use BNuT as an integrated data and communication system in our organization to better understand our practices and outcomes in home health and to lead and manage our organization proactively*.

[CEO Home Health Organization]

Competing institutional priorities further hindered progress. Post-COVID recovery, ongoing EHR upgrades, and accreditation audits absorbed most organizational attention. Because BNuT did not have immediate regulatory or financial implications, it was consistently deprioritized. Organizational culture also played a role: hospitals with participatory cultures tended to show greater openness to collaboration, whereas highly hierarchical institutions rarely progressed beyond exploratory discussions. Overall, BNuT’s organizational challenges reflected the entrenched marginalization of nursing leadership within hospital governance, a systemic issue that extends beyond this specific innovation.

### Technological: Interoperability, Data Quality, and Usability

The technological domain presented the most concrete and persistent barriers. BNuT requires integration of about 250 data points, where approximately 150 data points can be drawn from existing systems: HRs, scheduling, EHRs, quality registries, and even locally maintained spreadsheets. Each organization used a unique constellation of software vendors and data formats, often without shared standards. Even when multiple hospitals used the same vendor, integration had to be redeveloped from scratch due to licensing and cost restrictions. Vendors frequently imposed additional charges for API access or custom data exports, and in several cases claimed that bespoke queries were required for every new request.

Manual preprocessing was initially necessary to conform to the BNuT data structure. Anticipating this challenge, the system was designed with a flexible, tiered integration architecture: organizations could upload data manually per data point through the web interface, push standardized files through an automated importer in an agreed-upon format, or connect directly to a PostgreSQL database structure into which raw data could be pushed with automated ETL processing. To further reduce this burden, the project contracted an external ETL specialist who configured direct connections to source systems where feasible. Despite this flexibility, the approach proved only partially effective due to heterogeneous database architectures, limited vendor documentation, and the cost of obtaining data exports from third-party software providers. Data quality issues were widespread: implausible values were common, and missingness varied widely between datasets. BNuT’s algorithms were designed to tolerate partial missingness, except for a few core variables essential for standardization, but the variability still limited analytic robustness. The persistence of these barriers, even given the system’s deliberately flexible architecture, underscores that the bottleneck was structural and rooted in vendor lock-in, fragmented data governance, and limited hospital IT capacity.

Despite these integration difficulties, the user-facing technology was well received. A professional user experience/user interface designer ensured that the interface was intuitive and visually clear, with a simplified radar chart summarizing 10 domains of team performance. Users could benchmark teams or explore subdomains with minimal training. The system was web-based (Software as a Service) and accessible through standard browsers, providing sufficient performance and stability for managerial use.

Maintenance and updates were provided by the research team in collaboration with a contracted developer. Hospitals were able to independently distribute surveys and upload local data, and feedback loops for technical improvement remained informal. Overall, the technical obstacles were concentrated in data extraction and integration rather than in software usability or performance.

A critical question arising from the BNuT experience is whether a model integrating approximately 250 data points was over-engineered. The breadth of the model reflects our conceptual ambition of capturing team balance across all domains identified as relevant in the empirical literature [9], an ambition that was codeveloped and endorsed by the participating organizations themselves. However, the practical consequence was a data-governance burden that exceeded the current capacity of most hospital sites, particularly those reliant on legacy systems, outsourced IT functions, or fragmented data architectures. Even the flexible integration mechanisms provided could not overcome the structural barriers imposed by vendor lock-in and the cost of data extraction from proprietary systems. This tension between comprehensiveness and feasibility represents a design challenge that is not unique to BNuT but characterizes many health information systems that attempt to bridge the gap between research-level measurement and operational reality [15]. Future iterations of such systems should consider adopting a modular architecture where a core set of indicators (operationalizable with minimal integration effort and immediately actionable for nursing managers) serves as the entry point, while the full model is reserved for organizations with sufficient digital infrastructure and data-governance capacity.

### Interdomain Fit: A Prerequisite for Sustainability

Analysis across HOT-fit domains highlights that implementation success depended on achieving simultaneous fit across all 3 dimensions. The nurse-led organization illustrated how a strong fit within the organizational domain, where nursing leadership held both strategic authority and resource control, could compensate for barriers in other areas. Once executive endorsement was secured, IT resources were allocated, data-sharing agreements expedited, and BNuT was embedded in strategic planning. The same technical hurdles that halted progress elsewhere became manageable. Conversely, where one domain lacked fit, most commonly technology, momentum in the others dissipated.

In several hospitals, delays in data integration eroded enthusiasm among nursing leaders who initially championed the tool. Similarly, where organizational structures prevented nursing leadership from influencing IT or financial decisions, human-level motivation could not translate into implementation capacity. Positive feedback loops only emerged when success was visible and endorsed from the top: early demonstrable value encouraged further engagement and resource allocation, but such cycles remained rare.

Overall, these patterns confirm the HOT-fit framework’s central proposition: the effectiveness of health information systems depends not merely on the functionality of individual components but on the equilibrium between them. Imbalance in any single domain propagates across the system, undermining overall fit. In BNuT’s case, technological constraints, particularly data integration and interoperability, acted as the principal bottleneck, yet organizational and cultural dynamics determined whether these constraints could be overcome. Strong leadership endorsement, clear strategic positioning of nursing within governance structures, and proactive attention to data-trust and communication dynamics proved essential conditions for sustaining progress. The nurse-led success case offers a critical insight: when nursing leadership holds both strategic authority and operational responsibility, implementation becomes feasible. In this organization, the same technical challenges that stalled progress elsewhere were systematically addressed because nursing leadership could directly allocate resources, prioritize IT projects, and embed BNuT within strategic planning. This demonstrates that the barriers encountered elsewhere were not primarily technical but organizational. Conversely, when nursing remains positioned as a cost-center with limited influence over budgets and technology decisions, even technically sound innovations may struggle to take root. Achieving fit across human, organizational, and technological systems is therefore not only a condition for digital success but also a reflection of the broader power balance between clinical professions and institutional structures within health care.

## Discussion

### Principal Findings

The main finding of this reflective analysis is that the implementation of BNuT succeeded only where simultaneous alignment across human, organizational, and technological domains was achieved, specifically in a nurse-led home health organization where leadership held both strategic vision and budgetary authority. Across the 7 hospital sites, adoption was prevented by converging barriers: data interoperability failures driven by vendor lock-in and fragmented IT architectures, restricted nursing leadership authority within hierarchical governance structures, and the substantial data-governance demands imposed by the system’s own breadth. These findings are not unique to the Belgian context but reflect structural dynamics widely documented in health informatics.

More specifically, the BNuT implementation illustrates how organizational complexity constrains decision-support adoption in nursing practice. Across sites, barriers arose primarily from misalignment between human, organizational, and technological domains, although BNuT’s own data requirements may also have exceeded the digital capacity of participating institutions. This finding echoes long-standing observations within implementation science that the success of health information technology depends less on technical soundness than on contextual fit within existing workflows, governance, and professional cultures [16].

The predominance of technological barriers parallels systematic reviews showing that interoperability failures remain the most persistent obstacle to decision-support integration [17,18]. As in those studies, the inability to access or standardize data across HR, scheduling, and clinical systems eroded perceived reliability and constrained analytic capability. These “barriers of insufficiency” [17] reflect structural deficits in hospital IT resourcing rather than isolated project limitations, reinforcing the need for policy-level interoperability mandates.

The vendor lock-in and data fragmentation observed in the BNuT project are not isolated to the Belgian health care context. Internationally, similar dynamics have been extensively documented. In England, the National Programme for IT (NPfIT) was ultimately dismantled in 2011, in part because of vendor lock-in and the inability to achieve interoperability across hospital trusts [19,20]. Subsequent NHS strategies have mandated open standards and fast health care interoperability resources–based interoperability, yet in practice, vendor-controlled data architectures continue to impede secondary use of clinical and workforce data [21-23]. In the United States, the 21st Century Cures Act introduced explicit information-blocking provisions to prevent vendors from restricting data access, reflecting federal recognition that proprietary data practices constitute a systemic barrier to innovation [24]. Within the European Union, the proposed European Health Data Space regulation represents an emerging attempt to establish cross-border interoperability standards that could, in principle, reduce the per-project negotiation costs that proved limiting in the BNuT experience [25]. Collectively, these international examples confirm that the barriers encountered by BNuT reflect a structural problem in health IT governance, and that no amount of tool-level design can compensate when system-level interoperability standards are absent.

At the organizational level, the imbalance between nursing leadership enthusiasm and restricted resource authority emerged as decisive. BNuT encountered resistance not from overt opposition but from structural dependency on nonclinical decision-makers, reflecting what has been described as the “negotiation of control” in health care technology implementation [26]. When nursing leaders lacked access to financial or technical levers, implementation stalled despite clinical support [27]. This pattern may reflect broader international challenges: research suggests that even within integrated health systems, resource allocation and workflow redesign remain chronic bottlenecks [28], while CNO authority limitations and IT resource allocation challenges appear to be documented across health care systems globally [17,27]. The single nurse-led organization in our sample, where strategic and budgetary authority were unified under nursing leadership, achieved full operational use, empirically supporting the HOT-fit premise that fit across all domains is a prerequisite for sustainability.

The human dimension was characterized by enthusiasm to hesitation, abstention to mistrust. Nurses did not resist technology per se, but distrusted implementations perceived as top-down or evaluative [29]. The anonymity of BNuT’s survey component mitigated some of these concerns, yet lingering fears of data misuse illustrate fragile power dynamics [26]. Time pressure and cognitive overload further limited engagement, with research showing that human-resource scarcity continues to undermine even well-designed interventions [17].

Taken together, our findings reinforce the view of health care organizations as complex adaptive systems in which technical, organizational, and human subsystems are tightly coupled [16]. Localized improvements in one domain cannot compensate for deficits in another. The nurse-led success case exemplifies this interdependence: strong executive sponsorship overcame technical and organizational inertia, producing a functioning sociotechnical equilibrium. In contrast, sites lacking such fit experienced cascading failure: data integration delays reduced managerial trust, which in turn decreased organizational priority. Implementation success thus required “all lights green,” confirming HOT-fit’s central premise that sustainable adoption depends on balanced interaction across domains.

The conceptual contribution of BNuT lies in extending decision support beyond patient-level staffing ratios towards team-level optimization, incorporating leadership, workload, and safety culture. This holistic approach addresses gaps identified in recent reviews of nursing decision-support systems, which report limited theoretical grounding and minimal postimplementation evaluation [30]. Yet the difficulties experienced in this project also underscore the persistent divide between research prototypes and operational systems. Bridging this divide will require not only improved technical integration but also structural empowerment of nursing leadership to act as equal partners in digital transformation.

The lessons from BNuT have concrete implications for the design of future nursing decision-support systems. First, scalability demands a shift from monolithic, all-or-nothing deployment towards modular architectures that allow organizations to adopt core functionality with minimal integration effort and progressively expand as digital maturity grows. An API-first, cloud-native design would reduce per-site configuration costs and enable integration with diverse IT ecosystems without requiring bespoke development for each vendor. Second, adaptability requires that indicator sets are configurable to organizational context: rather than imposing a fixed 250-variable model, future systems should allow institutions to select domains aligned with their strategic priorities and data availability, with the full model serving as an aspirational benchmark. Third, integrating predictive analytics, for example, flagging units where scheduled team composition falls below stability thresholds, or forecasting turnover risk based on employment pattern trends, would shift such tools from retrospective reporting towards prospective decision support, increasing their value for both unit-level workforce management and organization-wide strategic planning. Fourth, interoperability should be embedded by design rather than retrofitted: adopting open data standards and European Health Data Space–compatible data models from the outset would reduce dependency on vendor-specific export mechanisms. For clinical data, standards such as HL7 fast health care interoperability resources provide a foundation; however, workforce-specific data (including turnover, employment patterns, and team composition metrics) are not yet covered by existing interoperability standards, highlighting the need for standardized data models for nursing workforce analytics as a prerequisite for tools like BNuT.

Taken together, the findings demonstrate that sustainable digital innovation in health care depends on achieving fit across human, organizational, and technological systems. Where one domain fails, whether through technical fragmentation, limited leadership authority, or mistrust, momentum across the others may diminish. Conversely, fit across all domains enables small successes to compound into sustained adoption.

Furthermore, they reveal a troubling urgency paradox. Given mounting workforce shortages, cost pressures, and quality demands, strategic optimization of nursing team deployment should arguably be among health care organizations’ highest priorities. The nursing workforce represents both the highest operational cost and the most direct interface between an organization and its patients. Yet across the sites studied, workforce optimization tools were consistently deprioritized in favor of regulatory compliance systems, billing infrastructure, or projects with more immediate financial returns. This misalignment between stated workforce priorities and actual resource allocation warrants attention from nursing leadership and policymakers alike. Translating these insights into practice yields several actionable lessons for the key stakeholders shaping the future of the nursing workforce.

Several limitations of this viewpoint must be acknowledged. First, the authors were the developers of BNuT, which creates an inherent risk of presenting the tool more favorably than an independent analysis might. We have tried to apply a self-critical lens, particularly in examining whether the system’s own complexity contributed to adoption failure, but readers should interpret the analysis with this in mind. Second, the reflections presented here are grounded in 8 years of sustained stakeholder engagement rather than in formal qualitative data collection with recorded interviews, systematic coding, and member checking. This gives us considerable ecological breadth and depth of immersion, but lacks the methodological safeguards of structured qualitative research, and the insights may be subject to recall bias and selective interpretation. Third, all implementation attempts occurred within the Belgian health care system, with its specific configuration of hospital governance, vendor markets, and regulatory frameworks. Although we have drawn parallels with international experiences, direct generalizability to other jurisdictions should not be assumed. Fourth, criterion validation of BNuT against patient and staff outcomes was planned but could not be completed because the sociotechnical barriers described in this paper prevented sustained implementation. The system’s effectiveness, therefore, remains empirically undemonstrated, and the tool should be understood as a theoretically informed prototype rather than a validated intervention. Fifth, the contrast between the single successful site and the 7 hospital sites should be interpreted with caution: the successful organization differed structurally in governance, scale, and patient population, and the comparison cannot isolate which specific factors were sufficient or necessary for adoption. Nonetheless, we believe that systematically documenting implementation failure through a structured analytical framework serves an important function in the health informatics literature, where publication bias towards successful cases limits collective learning.

### Lessons Learned

The synthesis of our findings, summarized in Figure 2, yields actionable lessons for the diverse stakeholders involved in digital nursing innovation. While grounded in the BNuT experience, these insights reflect broader structural issues relevant to sociotechnical implementation in health care.

#### Researchers

For researchers developing digital decision-support tools, the BNuT case offers a sobering lesson. BNuT was developed bottom-up from explicit practitioner demand: nursing leaders requested better decision-support tools, and 18 health care organizations collectively crowdfunded its development. The project incorporated end user co-design throughout, with nursing managers and CNOs involved in iterative development cycles. Implementation science principles guided the approach, including stakeholder mapping, staged rollout, and continuous evaluation. Yet despite these methodological strengths, BNuT still encountered fundamental barriers that prevented adoption in most sites. This suggests that the obstacles to data-driven decision support in nursing may be more systemic than methodological: even well-designed, practitioner-endorsed innovations may fail when organizational structures do not grant nursing leadership the authority to implement them. Researchers should therefore embed evaluation within a sociotechnical framework from the outset, document contextual mechanisms rather than merely outputs, and advocate for the structural conditions that enable adoption, not only develop technically sound tools.

#### Health Care Organizations

For health care organizations, our analysis underlines that the success of nursing informatics projects depends on granting nursing leadership formal authority over budget and data infrastructure. CNO enthusiasm alone cannot compensate for siloed governance or outsourced IT functions where access to the health care organization’s own data is locked by software vendors. Health care organizations need to address this issue in future contract negotiations with the software vendor so that access to their proper data can be faster, more flexible, and at a much lower additional cost. Organizations that positioned nursing as an equal partner in strategic planning achieved smoother integration and sustained use. Embedding nursing analytics tools such as BNuT within long-term workforce strategies, rather than treating them as stand-alone one-fits-all pilots, fosters alignment across organizational hierarchies and improves accountability.

#### Policymakers

For policymakers, the BNuT experience highlights that the barriers to digital nursing innovation are systemic rather than local. Fragmented data architectures, vendor lock-in, and inconsistent interpretations of data-protection regulation create environments where interoperability remains prohibitively expensive. Concretely, this requires policy action on multiple fronts. First, mandatory interoperability standards for health IT vendors would reduce the per-project negotiation costs that limited BNuT’s data integration. Second, dedicated pilot funding mechanisms for nurse-led digital health innovation could provide the financial continuity that free proof-of-concept approaches cannot sustain. The BNuT experience suggests that offering a tool free of charge may paradoxically reduce organizational commitment; phased funding models tied to implementation milestones might better incentivize sustained engagement. Third, governance reforms that mandate nursing representation on hospital IT steering committees and digital transformation boards would address the chronic underrepresentation of nursing leadership that proved decisive across our implementation sites. Addressing these “barriers of insufficiency” [17] requires structural investment in digital infrastructure and governance. Equally essential is embedding nursing leadership within national digital health strategies. Policies that enable nurse leaders to access and use workforce data are not merely equity measures but economically rational interventions that enhance quality, safety, and system performance.

### Conclusions

The BNuT experience demonstrates that the challenge of digital transformation in nursing may lie less in technology than in achieving fit across sociotechnical domains. Sustainable innovation appears to depend on the concurrent readiness of human, organizational, and technological systems, none of which can substitute for another. When fit is achieved, as in the nurse-led organization, digital tools can become enablers of strategic decision-making rather than isolated projects. The persistent barriers encountered across most sites reflect structural issues that extend far beyond any single system: the undervaluation of nursing leadership, fragmented data infrastructures, and chronic underinvestment in interoperability. Equally, our experience suggests that decision-support systems of this scope must be designed with explicit attention to the data-governance capacity of target organizations, adopting modular architectures that lower the threshold for initial adoption. Addressing these systemic imbalances—in interoperability governance, nursing leadership authority, and the design of implementation-ready tools—will be essential not only for future iterations of BNuT but for the broader success of nursing informatics as a driver of resilient and learning health care organizations.

## Abbreviations

API: application programming interface
BNuT: balanced nursing teams
CNO: Chief Nursing Officer
EHR: electronic health record
HOT-fit: Human-Organization-Technology fit
HR: human resource
NPfIT: National Programme for IT

## References

[1] Galanis P, Moisoglou I, Katsiroumpa A (2023). Increased job burnout and reduced job satisfaction for nurses compared to other healthcare workers after the COVID-19 pandemic. Nurs Rep.

[2] Haegdorens F, Van Bogaert P, De Meester K, Monsieurs KG (2019). The impact of nurse staffing levels and nurse’s education on patient mortality in medical and surgical wards: an observational multicentre study. BMC Health Serv Res.

[3] Aiken LH, Sloane DM, Bruyneel L (2014). Nurse staffing and education and hospital mortality in nine European countries: a retrospective observational study. Lancet.

[4] Ausserhofer D, Zander B, Busse R (2014). Prevalence, patterns and predictors of nursing care left undone in European hospitals: results from the multicountry cross-sectional RN4CAST study. BMJ Qual Saf.

[5] Drennan VM, Ross F (2019). Global nurse shortages-the facts, the impact and action for change. Br Med Bull.

[6] Shembavnekar N, Buchan J, Bazeer N (2022). REAL centre projections. https://www.health.org.uk/sites/default/files/2022-07/REALCentreWorkforceProjections_EMBARGOED.pdf.

[7] Ying LY, Ramoo V, Ling LW (2021). Nursing practice environment, resilience, and intention to leave among critical care nurses. Nurs Crit Care.

[8] Bae SH (2022). Noneconomic and economic impacts of nurse turnover in hospitals: a systematic review. Int Nurs Rev.

[9] Vleminckx S, Van Bogaert P, De Meulenaere K, Willem L, Haegdorens F (2024). Factors influencing the formation of balanced care teams: the organisation, performance, and perception of nursing care teams and the link with patient outcomes: a systematic scoping review. BMC Health Serv Res.

[10] Griffiths P, Saville C, Ball J (2020). Nursing workload, nurse staffing methodologies and tools: a systematic scoping review and discussion. Int J Nurs Stud.

[11] Yusof MM, Paul RJ, Stergioulas LK (2006). Towards a Framework for Health Information Systems Evaluation.

[12] Bogaert P, Clarke S (2017). The Organizational Context of Nursing Practice: Concepts, Evidence, and Interventions for Improvement.

[13] (2024). MAHA-sectoranalyse. Belfius Bank.

[14] Belfius Bank (2025). MAHA-sectoranalyse.

[15] Greenhalgh T, Wherton J, Papoutsi C (2017). Beyond adoption: a new framework for theorizing and evaluating nonadoption, abandonment, and challenges to the scale-up, spread, and sustainability of health and care technologies. J Med Internet Res.

[16] Sittig DF, Singh H (2010). A new sociotechnical model for studying health information technology in complex adaptive healthcare systems. BMJ Qual Saf.

[17] Chen W, O’Bryan CM, Gorham G (2022). Barriers and enablers to implementing and using clinical decision support systems for chronic diseases: a qualitative systematic review and meta-aggregation. Implement Sci Commun.

[18] Abell B, Naicker S, Rodwell D (2023). Identifying barriers and facilitators to successful implementation of computerized clinical decision support systems in hospitals: a NASSS framework-informed scoping review. Implement Sci.

[19] Campion-Awwad O, Hayton A, Smith L, Vuaran M (2014). The National Programme for IT in the NHS. A case history. https://www.alejandrobarros.com/wp-content/uploads/2019/07/Historia-Proyecto-TI-NHS.pdf.

[20] Wachter R (2016). Making IT work: harnessing the power of health information technology to improve care in England. https://rpsg.org.uk/wp-content/uploads/2020/04/Technology-to-Improve-Care-in-England.pdf.

[21] Bennion MR, Spencer R, Moore RK, Kenyon R (2025). Digital capability, open-source use, and interoperability standards within the national health service in England: survey of health care trusts. JMIR Hum Factors.

[22] Cresswell K, Sheikh A, Krasuska M (2019). Reconceptualising the digital maturity of health systems. Lancet Digit Health.

[23] Department of Health and Social Care (2022). Data saves lives: reshaping health and social care with data. HM Government.

[24] Adler-Milstein J, Pfeifer E (2017). Information blocking: is it occurring and what policy strategies can address it?. Milbank Q.

[25] Terzis P, Santamaria Echeverria OE (2023). Interoperability and governance in the European Health Data Space regulation. Med Law Int.

[26] Liberati EG, Ruggiero F, Galuppo L (2017). What hinders the uptake of computerized decision support systems in hospitals? A qualitative study and framework for implementation. Implement Sci.

[27] Alexander C, Tschannen D, Argetsinger D, Hakim H, Milner KA (2022). A qualitative study on barriers and facilitators of quality improvement engagement by frontline nurses and leaders. J Nurs Manag.

[28] Shi Y, Amill-Rosario A, Rudin RS (2021). Barriers to using clinical decision support in ambulatory care: do clinics in health systems fare better?. J Am Med Inform Assoc.

[29] Pareek A, Soni H, Bisen A (2024). Nursing informatics adoption in clinical settings: overcoming barriers and leveraging facilitators. Universe Int J Interdiscip Res.

[30] Abi Khalil C, Saab A, Rahme J, Bouaud J, Seroussi B (2025). Capabilities of computerized decision support systems supporting the nursing process in hospital settings: a scoping review. BMC Nurs.

